# The evolution and genomic landscape of *CGB1* and *CGB2* genes

**DOI:** 10.1016/j.mce.2005.11.049

**Published:** 2007-01-02

**Authors:** Pille Hallast, Kristiina Rull, Maris Laan

**Affiliations:** a Department of Biotechnology, Institute of Molecular and Cell Biology, University of Tartu, Riia 23, 51010 Tartu, Estonia; b Department of Obstetrics and Gynecology, University of Tartu, Estonia

**Keywords:** Chorionic gonadotropin beta 1 and 2, Gene evolution, Polymorphism patterns, Putative promoter region, *In silico* transcription factor binding site prediction

## Abstract

The origin of completely novel proteins is a significant question in evolution. The luteinizing hormone (*LHB*)/chorionic gonadotropin (*CGB*) gene cluster in humans contains a candidate example of this process. Two genes in this cluster (*CGB1* and *CGB2*) exhibit nucleotide sequence similarity with the other *LHB*/*CGB* genes, but as a result of frameshifting are predicted to encode a completely novel protein. Our analysis of these genes from humans and related primates indicates a recent origin in the lineage specific to humans and African great apes. While the function of these genes is not yet known, they are strongly conserved between human and chimpanzee and exhibit three-fold lower diversity than *LHB* across human populations with no mutations that would disrupt the coding sequence. The 5′-upstream region of *CGB1*/*2* contains most of the promoter sequence of hCGβ plus a novel region proximal to the putative transcription start site. *In silico* prediction of putative transcription factor binding sites supports the hypothesis that *CGB1* and *CGB2* gene products are expressed in, and may contribute to, implantation and placental development.

## 1 Introduction

**Fig. 1:**
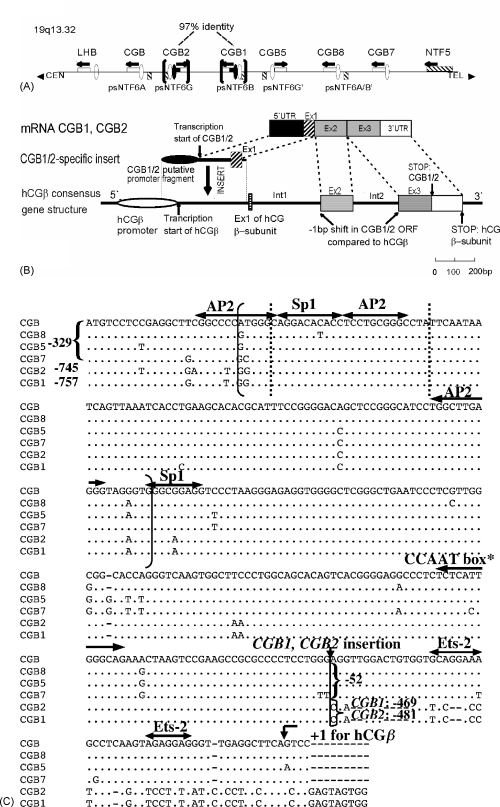

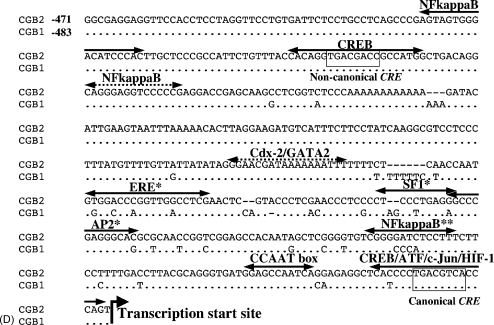
Genomic context of *CGB1* and *CGB2*. (A) Schematic presentation of the structure of the *LHB*/*CGB* gene cluster (covering 39.76 kb from *LHB* to *CGB7*) drawn to an approximate scale. Individual *LHB*/*CGB* genes (white boxes) cover 1.11–1.466 kb. Arrows indicate the direction of transcription either from a sense or an antisense strand. Experimentally identified hCGβ promoter sequence (Otani et al., 1988; white ovals) is also present, although more distally, upstream of *LHB*, *CGB1* and *CGB2* genes. Detailed alignment of the promoter area is shown in C. *CGB1* and *CGB2* specific insert is divided into a transcribed segment coding for 5′-UTR, exon1 and part of intron 1 of *CGB1*/*CGB2* (black boxes; 255 bp) and an immediate 5′-upstream segment, which could serve as an additional promoter component (black ovals; *CGB1* 481 bp, *CGB2* 469 bp). Alignment of the non-coding 5′-upstream part of the insert is in D. Intergenic Neutrophin 6 pseudogenes (*psNTF6*; striped boxes; <1.15 kb) originate through duplication from Neutrophin 5 (*NTF5*) exon 3 (Hallast et al., 2005). (B) Structure of *CGB1* and *CGB2* differs from a consensus hCGβ gene in the following aspects: (1) hCGβ 5′-UTR has been replaced by a *CGB1*/*2*-specific insert coding for *CGB1*/*2* 5′-UTR, exon 1 (diagonally striped box) and part of intron 1 (black box) as well as provides a 481/469 bp upstream fragment, which could function as an additional promoter segment (black oval); (2) hCGβ exon 1 (horizontally striped box) is a part of *CGB1/2* intron 1; (3) open reading frame (ORF) of exons 2 and 3 of *CGB1*/*2* (grey boxes) has a-1bp shifted compared to hCGβ coding genes; (4) shifted ORF has lead to earlier STOP codon and shorter exon 3. An alternative exon 1 and shifted ORF for exons 2 and 3 code for a putative CGB1/2 protein with no amino acid similarity to hCGβ-subunit. (C) Alignment of the proximal promoter of hCGβ subunit coding genes (*CGB*, *CGB5*, *CGB8*, *CGB7*) with the homologous upstream segment of *CGB1* and *CGB2*. cAMP response element has been mapped from −311 to −202 (Albanese et al., 1991; black brackets), trophoblast-specific element TSE from −305 to −279 (Steger et al., 1993; dotted brackets). Other experimentally proven regulatory elements of hCGβ promoter include activating protein 2 (AP2) and selective promoter factor 1 (Sp1) (Johnson and Jameson, 1999) as well as Ets-2 binding sites (Ghosh et al., 2003). ^*^CCAAT box has been identified by Matinspector and Alibaba TFBS prediction softwares. (D). Prediction of transcription factor binding sites (TFBS) onto the 5′-upstream segment unique to *CGB1* and *CGB2* created by the insertion (B). TFBSs predicted by both MatInspector and Alibaba methods are marked with solid arrows above the aligned sequences of *CGB1* and *CGB2*; TFBSs recognized by MatInspector alone are marked by broken arrows. TFBSs predicted solely based on *CGB1* sequence are indicated with (*) and based on *CGB2* (**). ATF: activating transcription factor; AP2: activating protein 2; Cdx2: Caudal-related transcription factor; CREB: cAMP responsive element binding protein; ERE: Estrogen response element; HIF: Hypoxia-inducible factor 1; NFkappaB: nuclear factor κB; GATA2: GATA-biding protein 2; SF1: steroidogenic factor 1; Sp1: selective promoter factor 1. Transcription start site has been indicated based on NCBI GenBank locus no NG_000019 information.

The human luteinizing hormone/chorionic gonadotropin beta (*LHB*/*CGB*) gene cluster on chromosome 19q13.3 consists of one *LHB* gene and six *CGB* genes (Fiddes and Goodman, 1980; Talmadge et al., 1984a; Maston and Ruvolo, 2002; Fig. 1A). These seven genes are highly conserved at the nucleotide level (85–99% DNA sequence identity) and appear to have originated from an ancestral *LHB* gene as a result of duplication during primate evolution. Four of the genes (*CGB*, *CGB5*, *CGB7* and *CGB8*) encode the beta subunit of human chorionic gonadotropin, a 163 amino acid protein that is produced by the implanting conceptus and is essential for alternations to the maternal reproductive system in support of pregnancy. The other *CGB* genes, *CGB1* and *CGB2*, encode a hypothetical protein of 132 amino acids that is completely different from the hCGβ-subunit and lacks similarity to any known protein (Bo and Boime, 1992). These genes appear to have evolved by insertion of a DNA fragment (736 bp for *CGB1*, 724 bp for *CGB2*) that replaces 52 bp of the proximal end of the promoter and the entire 5′-UTR of an ancestral hCGβ-subunit coding gene (Bo and Boime, 1992; Hollenberg et al., 1994; Fig. 1B). This insertion creates a *CGB1*/*CGB2* specific putative promoter fragment, an alternative 5′untranslated region (5′-UTR) and a novel exon 1, leading to a one basepair frameshift in the open reading frame (ORF) for exons 2 and 3.

Although a protein product corresponding to *CGB1* and *CGB2* has not yet been isolated, mRNA from these genes has been detected in the placenta (Bo and Boime, 1992; Rull and Laan, 2005) as well in the testis (Berger et al., 1994), pituitary (Dirnhofer et al., 1996), and in breast cancer tissue (Giovangrandi et al., 2001). The repeated observations of expression suggest that these genes are functional. In transgenic mice carrying a 36-kb cosmid insert with all the six *CGB* genes, the *CGB1* and *CGB2* transcripts were also observed in brain at levels comparable with placenta, the expression site for all the *CGB* genes (Strauss et al., 1993).

As the next step toward understanding the evolution and functional relevance of *CGB1* and *CGB2* we sequenced and analyzed the genes from three human populations as well as from the closest living relatives of humans. As a reference for considering relative conservation of *CGB1* and *CGB2* we used *LHB*, the founding member of this gene cluster and a gene that has a well-established, essential and conserved function in mammalian reproduction. We used the resulting data to explore the following questions: (i) what is the origin of *CGB1* and *CGB2*? (ii) how conserved are *CGB1* and *CGB2* among primates? (iii) does the variation pattern of human *CGB1* and *CGB2* support constraints on variation consistent with functionality? (iv) does the upstream region of human *CGB1*/*2* have expected features of a functional promoter and what transcription factor binding sites are present that could direct expression of these genes to specific tissues?

## 2 Materials and methods

### 2.1 Experimental subjects

The study was approved by the Ethics Committee of the University of Tartu, Estonia (protocol no. 117/9, 16 June 2003). *CGB1* and *CGB2* genes were resequenced for 47 Estonian (Europe), 23 Mandenka (Africa) and 25 Chinese Han (Asia) individuals. Estonian DNA samples originate from the DNA bank of Department of Biotechnology, IMCB, University of Tartu. Mandenka and Han DNA samples were obtained from HGDP-CEPH Human Genome Diversity Cell Line Panel (http://www.cephb.fr/HGDP-CEPH-Panel/). Common chimpanzee (*Pan troglodytes*) DNA was extracted from sperm material obtained from Tallinn Zoo, Estonia; sources of orangutan (*Pongo pygmaeus*) and gorilla (*Gorilla gorilla*) DNAs were primary cell lines AG12256 and AG05251B, purchased from ECACC.

### 2.2 Sequencing of human and great ape *CGB1* and *CGB2* genes

The structure of the *CGB1*/*CGB2* (#MIM, 608823; 608824) genomic region has been reconstructed by web-based global alignment (http://www.ebi.ac.uk/clustalw/;CLUSTALW) and BLAST (http://www.ncbi.nlm.nih.gov/BLAST/) tools. For the analysis we used the sequence obtained from NCBI GenBank database (http://www.ncbi.nlm.nih.gov; locus no NG_000019; 26 June 2002 release).

PCR and sequencing primers for *CGB1*, *CGB2* and a reference gene *LHB* were designed based on human sequence using the web-based version of the Primer3 software (http://frodo.wi.mit.edu/cgi-bin/primer3/primer3_www.cgi; Table 1). To guarantee the unique amplification of *CGB1* and *CGB2*, one of the primers was located within the most divergent segments of *CGB1*/*2*-specific insertion region. The uniqueness of all PCR primer pairs was checked using BLAST. The genes were amplified to cover the entire coding sequence and part of flanking regions (for human *CGB1* 1600 bp; *CGB2* 1652 bp; *LHB* 1599 bp PCR products). Primers designed on human sequence were also used to amplify *CGB1* and *CGB2* as well as *LHB* from genomic DNA of common chimpanzee, gorilla and orangutan. However, in order to overcome possible divergence among the species, we used a panel of primers and primer combinations (Table 1). In total eight PCR primer combinations were tested for *CGB1* and nine for *CGB2* amplification from chimpanzee, gorilla and orangutan DNA. PCR amplification of 100 ng genomic DNA (Long PCR Enzyme Mix; MBI Fermentas) was performed in a PTC-200 thermal cycler (MJ Research) using a standard protocol recommended by the manufacturer. The reactions were initiated with a denaturation at 95 °C for 5 min, followed by 10 cycles of denaturation at 95 °C for 20 s, annealing at 68 °C for 30 s (decrease of temperature 1 °C per cycle), elongation at 68 °C for 2 min, 10 cycles 95 °C (20 s), 56 °C (30 s), 68 °C (2 min), 10 cycles 95 °C (20 s), 54 °C (30 s), 68 °C (2 min), 10 cycles 95 °C (20 s), 51 °C (30 s), 68 °C (2 min). A final extension step was performed at 68 °C for 10 min.

All amplified genes were sequenced from both strands. For removing unincorporated mononucleotides and PCR primers, PCR products were treated with shrimp alkaline phosphatase (1.5 U, USB) and exonuclease I (1 U, MBI Fermentas). Incubation was performed in a GeneAmp^®^ PCR System 2700 thermal cycler (Applied Biosystems) at 37 °C for 20 min followed by enzyme inactivation at 80 °C for 15 min. Purified PCR product (1.5–3 μl) was used as a template in sequencing reactions (10 μl) along with a sequencing primer (2 pmol) and DYEnamic ET Terminator Cycle Sequencing Kit reagent premix (Amersham Biosciences Inc.) as recommended by the supplier. Sequencing reactions (1.5 μl) were resolved on ABI 377 Prism automated DNA sequencer (Applied Biosystems) using ReproGel™ 377 gels (Amersham Biosciences Inc.). Genes of great apes were sequenced with both human-specific as well as species-specific primers designed by primer-walking approach. Sequencing primers are listed in Table 1.

The sequence data was assembled into a contig using phred and phrap software, the contig was edited in consed package (http://www.phrap.org/phredphrapconsed.html). Human polymorphisms were identified using the polyPhred program (Version 4.2) (Nickerson et al., 1997) and confirmed by manual checking. Allele frequencies of identified human SNPs were estimated and conformance with Hardy–Weinberg equilibrium was computed by an exact test (*α* = 0.05) using HaploView (Barrett et al., 2005) program.

Alignment of human and great apes *CGB1* and *CGB2* genomic sequences was performed by web-based global alignment tool CLUSTALW (http://www.ebi.ac.uk/clustalw/).

### 2.3 Sequence diversity parameters and neutrality tests

Sequence diversity parameters were calculated by DnaSP software (Version 4.0) (Rozas and Rozas, 1999). The direct estimate of per-site heterozygosity (*π*) was derived from the average pairwise sequence difference, and Watterson's *θ* (Watterson, 1975) represents as an estimate of the expected per-site heterozygosity based on the number of segregating sites (S). Tajima's D (*D*^T^) statistic (Tajima, 1989) was performed to determine if the observed patterns of human *CGB1*, *CGB2* and a reference *LHB* gene diversity are consistent with the standard neutral model. The basis of *D*^T^ value is the difference between the *π* and *θ* estimates: under neutral expectation *π* = *θ* and *D*^T^ = 0. Significant positive *D*^T^ values indicate an excess of intermediate-frequency alleles in a population consistent with either balancing selection or population bottleneck, whereas significant negative *D*^T^ values indicate an excess of rare SNPs consistent with either recent directional selection or an increase in population size.

A simple neutral model (Kimura, 1983) predicts that drift and mutation rate determine the level of nucleotide variation accumulating within and between species. Therefore, the relative amount of within-species polymorphism should reflect the amount of between-species fixation under neutrality. Genetic diversity of human *CGB1* and *CGB2* was compared with fixation between human and chimpanzee, as well as human and gorilla sequences to test neutrality. We applied the Hudson, Kreitman and Aguade (HKA) test (Hudson et al., 1987) to estimate whether there was a significant difference in the ratio of polymorphism to divergence of across *CGB1* and *CGB2* using *LHB* as a reference locus.

### 2.4 In silico prediction of TFBS to human *CGB1* and *CGB2* 5′-upstream region

Prediction of transcription factor binding sites (TFBS) was performed using the MatInspector 2.2 (http://www.genomatix.de/products/MatInspector/; Cartharius et al., 2005) and Alibaba 2.1 (http://www.gene-regulation.com/pub/programs/alibaba2/index.html; Grabe, 2002) programs. Both approaches rely on the information about the experimentally defined TFBS collected in the TRANSFAC database (http://www.gene-regulation.com/pub/databases.html#transfac; Matys et al., 2003). MatInspector identifies TFBS in nucleotide sequences using a large library of position weight matrices (PWM). PWM is a common way to represent the degenerate sequence preferences of a DNA-binding protein (reviewed by Stormo, 2000). Briefly, the elements of PWM correspond to scores reflecting the likelihood that particular nucleotide at the particular position can be observed as the known or candidate TFBS. A weight matrix pattern definition is superior to a simple IUPAC (International Union of Pure and Applied Chemistry) consensus sequence as it represents the complete nucleotide distribution of each single position. It also allows the quantification of the similarity between the weight matrix and a potential TFBS detected within the sequence. Alibaba program starts directly at the known binding sites instead of using predefined matrices in the database. The analysis is a process consisting of three steps: (1) it pairwise aligns of known sites to the unknown sequence; (2) it forms small sets of sites by their position and their according class of factor; (3) it constructs matrices from these sets. We run the Alibaba 2.1 under the following conditions: Pairsim to known sites 64, matrix width 10 bp, minimum number of sites 4–5, minimum matrix conservation 75%, similarity to sequence matrix 1%, factor class level 4–5.

When the reliability of the methods was tested on the previously determined *CGB5* promoter (Otani et al., 1988; Albanese et al., 1991; Steger et al., 1993; Hollenberg et al., 1994; Fig. 1C), MatInspector algorithm predicted one of the two experimentally proved (Johnson and Jameson, 1999) AP2 binding sites and two of three Sp-sites as well as CCAAT and CG-boxes, while Alibaba approach was capable of recognizing correctly one Sp1 site and CCAAT box. In the subsequent analysis of *CGB1*/*2*-specific upstream fragment, we relied predominantly on predictions by MatInspector approach.

## 3 Results and discussion

### 3.1 *CGB1* and *CGB2* have possibly arisen in the common ancestor of African great apes

First, we addressed the question of conservation of *CGB1* and *CGB2* genes among the species. Human-specific primers were used to amplify a unique gene product of *CGB1* for chimpanzee (primers CGB1_2F and CGB1_6R, predicted length based on human sequence 2312 bp; Genbank accession no. DQ238547) and of *CGB2* for gorilla (CGB2_1F and CGB2_3R, 1812 bp; DQ238550). Chimpanzee *CGB2* was inferred from the jointly amplified *CGB1*/*2* products (primers CGB2_1F and CGB2_5R, 2269 bp; DQ238549) using the chimpanzee *CGB1* sequence as a reference. With a similar approach, the gorilla *CGB2* was used as a reference to derive *CGB1* from a common *CGB1*/*2* product amplified from gorilla DNA (primers CGB1_1F and CGB1_2R, 1600 bp; DQ238548). None of the human-specific *CGB1* and *CGB2* primer combinations were capable to amplify the expected products from orangutan genomic DNA. Therefore, either the orangutan *CGB1* and *CGB2* sequences are highly divergent from other studied primates; or this species lacks *CGB1*/*2* insertion region (target of one of the primers), and consequently *CGB1*/*2* genes. The latter scenario is also supported by a recent study suggesting the total copy number of orangutan *CGB* genes to be four (Maston and Ruvolo, 2002). Consequently, we raise the hypothesis of the origin of *CGB1* and *CGB2* in the common ancestor of African great apes.

Amplification of the reference gene *LHB*, a functional ancestral member of the same gene cluster was successful with human-specific primers in all four studied species: human, chimpanzee (Genbank accession no. DQ238551), gorilla (DQ238552) and orangutan (DQ238553).

### 3.2 *CGB1* and *CGB2* are conserved between human and chimpanzee

**Fig. 2:**
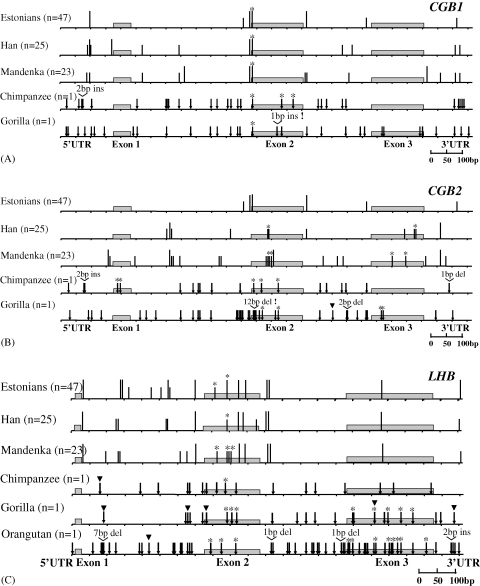
SNP patterns and fixed differences between human and great apes in *CGB1* (A), *CGB2* (B) and *LHB* (C) genes. Human polymorphic positions (vertical black bars) are marked as long bars for common SNPs (minor allele frequency >10%) and short bars for rare SNPs (<10%). For human and great ape comparison fixed differences (black arrows), non-synonymous changes (black arrows with an asterisk), SNPs found in apes (black triangle) and protein altering insertions/deletions are shown (exclamation mark).

Divergence of chimpanzee (C) and gorilla (G) *CGB1*, *CGB2* and *LHB* from human (H) sequences (Table 2; H/C: across the genes 1.35–2.19%, exons 0.5–1.42%, introns 1.53–2.68; H/G: across the genes 1.44–3.00%, exons 1.42–4.04%, introns 1.36–3.31%) somewhat exceeds previous estimations. The average divergence across 53 autosomal intergenic regions has been reported 1.24 ± 0.07% for H/C and 1.62 ± 0.08% for H/G (Chen and Li, 2001). Human/chimpanzee comparison of 127 genes mapped to human chr. 21 resulted in estimates of overall divergence for coding sequences 0.75 ± 0.01% (range 0.53–2.05%), for exons 0.51% ± 0.02 (range 0.08–2.52%), for exon/intron junction 0.85 ± 0.02% (range 0.41–2.78%) for 5′-UTR 1.00% ± 0.10 and for 3′-UTR 0.93% ± 0.09 (Shi et al., 2003). Relatively high divergence (across the gene 5.39% compared to 3.08% reported for intergenic regions; Chen and Li, 2001) was also estimated between human and orangutan (O) for *LHB* including 11 non-synonymous changes. Higher interspecific divergence could result from the intraspecific gene conversion among highly homologous genes in the *LHB*/*CGB* cluster (Maston and Ruvolo, 2002; Hallast et al., 2005). For gorilla *CGB2* gene approximately two fold higher sequence divergence for H/G compared to H/C largely arises from two gorilla-specific deletions (2 and 12 bp) increasing substantially the number of fixed nucleotide differences between species (Fig. 2; supplementary figure).

Divergence patterns between human and chimpanzee *CGB1* and *CGB2* resemble the reference gene *LHB*, characterized by higher conservation in exons compared to introns (Table 2). We identified only a few fixed differences among species causing non-synonymous changes in chimpanzee *CGB1* (3), *CGB2* (5) and *LHB* (1) relative to human sequence (Fig. 2; supplementary figure). None of the sequence differences alter the ORF nor create a preliminary stop-codon. The evolution of 5′- and 3′-UTR sequences is variable among the genes, from 0 differences to 3.4% divergence. In gorilla the overall number of non-synonymous changes is even higher for *LHB* (8) than for *CGB1* (1) and *CGB2* (4). However, in gorilla we identified for both *CGB1* (1 bp insertion in exon 2) and *CGB2* (12 bp deletion at the beginning of exon 2 removing an Ala-Val-Ala-Ala motif) a change presumably leading to the disruption of a predicted protein (Fig. 2). Whether these represent consensus sequences for gorilla *CGB1* and *CGB2*, or only mutations in the genome of the sequenced individual will be solved when additional gorilla sequences are available for comparison.

In summary, the interspecific analysis of *CGB1* and *CGB2* indicates that the level of conservation between human and chimpanzee is as high as for *LHB*, thus supporting the functional importance of these genes in these species. However, in gorilla the functionality of *CGB1* and *CGB2* is less likely as disrupted ORF was identified for *CGB1* and a large deletion in exon 2 for *CGB2*.

### 3.3 Resequencing of human *CGB1* and *CGB2* genes revealed low variation and no nonsense mutations

As a next step we studied the polymorphism patterns of human *CGB1* and *CGB2* in comparison to *LHB* gene. Re-sequencing of total 190 chromosomes from three human populations (Estonians *n* = 94, Chinese Han *n* = 50 and Mandenka *n* = 46) identified 22 single nucleotide polymorphisms (SNPs) in *CGB1* (ss48399944*–*ss48399963), 30 in *CGB2* (ss48399964*–*ss48399997) and 24 in *LHB* (ss48399882*–*ss48399908) (Fig. 2; supplementary table). Interestingly, the *LHB* gene exhibited even three-fold higher variation than *CGB1* and *CGB2* (Table 3; average across populations: *π*_LHB_/kb = 3.92; *π*_CGB1_/kb = 1.39; *π*_CGB2_/kb = 1.26). Only one polymorphism in *CGB1*, five in *CGB2* and four in *LHB* were identified leading to a non-synonymous change. None of the polymorphisms found in coding regions created a preliminary stop codon. Thus, the diversity patterns of human *CGB1* and *CGB2* comparable with a typical variation of human genes (African Americans *π*_(74 genes)_/kb = 1.00; European Americans *π*_(74 genes)_/kb = 0.80; Crawford et al., 2004) and rare non-synonymous substitutions give support to the functionality of these genes. Identification of ancestral alleles of human SNPs in comparison with other great ape sequences revealed that for most of the SNPs the major allele in human is also the ancestral variant (supplementary table).

We performed two alternative analyses to test whether the human *LHB*, *CGB1* and *CGB2* have evolved under standard neutral model. Tajima's test examines whether the average number of pairwise nucleotide differences between sequences (*π*) is larger than expected from the observed number of polymorphic sites (*θ*). The expected difference (Tajima D) between *π* and *θ* is roughly zero under the standard neutral model. As differences between *π* and *θ* for the studied genes were small and Tajima D values were close to zero (Table 3), the hypothesis of neutral evolution of these genes was not rejected. The HKA test was performed to test the neutral evolution of *CGB1* and *CGB2* among the studied species with *LHB* as a reference (Table 4). The test is based on prediction from the Neutral Theory of Molecular Evolution (Kimura, 1983) that the amount of within-species diversity should be correlated with levels of between-species divergence, due to the dependence of both on the neutral mutation rate. Consistently with Tajima's test, we could not reject the null hypothesis of neutral evolution. The exception was the Estonian sample set, where the significant result could be spurious outcome of the population history in Europe shaped by bottlenecks (Barbujani and Goldstein, 2004) and thus capturing the least the human intrapopulation variation applied in HKA test. However, as neither Tajima's nor HKA test takes into account gene conversion shown to shape the sequences and variation of *LHB*/*CGB* genes within a species (Maston and Ruvolo, 2002; Hallast et al., 2005), we should interpret the overall test results with caution and could not entirely exclude selection. Innan (2003) has shown that statistical tests of neutrality based on the standard coalescent theory for a single-copy gene may not be appropriate for duplicated genes.

### 3.4 *CGB1* and *CGB2* genes possess almost complete hCGβ promoter sequence

In order to predict the regulatory elements and patterns putatively involved in driving the expression of *CGB1* and *CGB2*, we investigated the upstream regions of these genes. Alignment of the experimentally identified hCGβ promoter (−311 bp from hCGβ 5′-CAP; Otani et al., 1988; Hollenberg et al., 1994) with the 5′-upstream region of *CGB1* and *CGB2* genes revealed a more proximal location of an almost complete hCGβ promoter sequence (−757 to −481 for *CGB1* and −745 to −469 for *CGB2* from predicted transcription start site), lacking only 52 bp of proximal promoter segment of hCGβ (Fig. 1B and C). Despite the absence of the minimal promoter region (MPR; −37 to +104; Hollenberg et al., 1994) including two Ets-2 binding sites (Ghosh et al., 2003), the other sequence motifs playing a crucial role in regulating hCGβ expression are conserved among the genes (Fig. 1C). These include cAMP-dependent transcription element mapped to −311 to −202 (Albanese et al., 1991), trophoblast-specific element (TSE) between −305 and −279 maintaining basal expression (Steger et al., 1993), as well as binding sites for AP-2 and Sp1 transcription factors required for the full activity of the promoter (Johnson et al., 1997; Johnson and Jameson, 1999; Knöfler et al., 2004). Hollenberg et al. (1994) has suggested that the individual domains of the hCGβ promoter act in an addictive or combinatory manner. Thus, the absence of hCGβ MPR from *CGB1*/*2* putative promoter region could possibly be compensated by the sequences within *CGB1*/*2*-specific insertion. However, whether this segment indeed has a regulatory function for *CGB1*/*2* needs to be proven in wet-lab experiments.

### 3.5 *CGB1*- and *CGB2*-specific upstream region is predicted to harbor binding sites for transcription factors related to early placental development and implantation

In addition to alternative 5′-UTR and exon 1 for *CGB1* and *CGB2* genes, the *CGB1*/*2*-specific insert (736 and 724 bp, respectively) provides a novel putative proximal promoter segment (481 and 469 bp, respectively) upstream the transcription start site (Fig. 1B). We evaluated this fragment as a potential additional promoter component. *In silico* analysis predicted several regulatory elements in *CGB1*/*2* insert experimentally determined to be essential for gonadotrope expression (Fig. 1D): two copies of CRE sites binding cAMP-responsive element binding protein (CREB) and activating transcription factor (ATF), binding sites for CGα and CGβ transcription inducer AP-2 as well as for repressor c-Jun (Pestell et al., 1994; Johnson et al., 1997), binding sites for GATA-2 regulating CG α-subunit expression in placenta (Steger et al., 1994).

Interestingly, the *CGB1*/*2* specific upstream region is predicted to harbor interaction sites for several transcription factors regulating implantation and placental development (Fig. 1D): NF-κB, Cdx-2, ERR-β, HIF1 and SF-1. Although NF-κB is a transcriptional factor involved mainly in inflammatory and immune responses, it regulates also several genes responsible for immunological adaptation at the feto-maternal interface and early embryonic development (Chen et al., 1999; Muggia et al., 1999). Both, in human (Page et al., 2002) and mouse (Nakamura et al., 2004) NF-κB is activated in the pregnant uterus during preimplantation period and is highly expressed during the implantation window.

Cdx-2 and ERR-β exhibit highly specific expression pattern during embryogenesis. Besides its main role in driving embryonic axial elongation and anterior–posterior patterning, Cdx2 is also essential for trophoblastic development (Chawengsaksophak et al., 2004). Consistently, aberrant expression of bovine *Cdx2* in the preimplantation cloned embryo has been reported to lead to the failure of implantation (Hall et al., 2005). ERE (reviewed by Gruber et al., 2004) is a binding site (consensus sequence 5′-GGTCAnnnTGACC-3′) not only for the estrogen receptor ligand complex, but also for ERR-β, an orphan member of the superfamily of nuclear hormone receptors (Pettersson et al., 1996). Studies on mice have shown that ERR-β is expressed during embryogenesis by ectodermally derived regions of the amniotic fold, forming chorion. Homozygous mutant mouse embryos generated by targeted disruption of the *Estrrb* gene have severely impaired placental development, and die 10.5 days post-coitum (Luo et al., 1997).

Hypoxia-inducible factors (HIFs) mediating oxygen homeostasis have been suggested to regulate uterine vascular permeability and angiogenesis (Daikoku et al., 2003). Transcription factor SF-1 is a key regulator of the transcription of many genes involved in sexual differentiation, steroidogenesis and reproduction (*GnRHR*, *α-GSU*, *FSHB* and *LHB*; reviewed by Parker and Schimmer, 1997).

*In silico* prediction of putative transcription factor binding sites allows to postulate the hypothesis of the involvement of *CGB1* and *CGB2* gene products in implantation and placental development. The hypothesis is supported by the detection of *CGB1* and *CGB2* transcripts in the placenta, although at much lower level compared to hCGβ coding genes (Bo and Boime, 1992; Rull and Laan, 2005).

## 4 Conclusions

This report aimed to explore the evolution, variation and putative regulatory regions of *CGB1* and *CGB2* in order to seek indirect evidence for the functionality of these genes, originally considered as pseudogenes (Talmadge et al., 1984b).

As both of the genes were amplified additionally to human also in chimpanzee and gorilla but not in orangutan, we suggest that they have arisen among the common ancestor of African great apes. Gene duplication was accompanied by the replacement of the hCGβ 5′-UTR with a non-coding sequence providing novel putative promoter segment, 5′-UTR and exon 1.

In human, *CGB1* and *CGB2* exhibit three times lower diversity than for *LHB* and no ORF disturbing mutations for a sample representing three continents. Both genes are conserved between human and chimpanzee, exhibiting the same level of interspecific divergence as *LHB*. Especially *CGB1* stands out with a strong exonic conservation with only 0.5% divergence between human and chimpanzee, whereas the respective number for *LHB* is 1.42%. In contrast, for gorilla both *CGB1* and *CGB2* harbor insertion/deletion changes, which disrupt the predicted protein and thus there is little support for the functionality of these genes. We hypothesize that the fate of duplicated *CGB1* and *CGB2* genes has split for human–chimpanzee and gorilla lineages evolving towards a novel functional gene for the former and pseudogenization for the latter.

Upstream *CGB1* and *CGB2* is preserved almost full and well conserved (among genes) sequence of the promoter for hCGβ coding genes. Additionally, *CGB1*/*2* possess a novel putative proximal promoter segment created by the *CGB1*/*2*-specific insertion. Analysis of this segment *in silico* for TFBSs highlighted several elements shown to regulate gene expression during implantation and placental development. However, as TFBS prediction programs can only infer the binding potential, and not the functionality of the site, only succeeding wet-lab experiments are able to uncover whether the predictions and postulated hypothesis hold true.

## Figures and Tables

**Table 1:** Primers used for human and great ape PCR amplification and DNA sequencing

Gene	Primers	Sequence 5′–3′
Human-specific PCR primers-pairs		
*CGB1*	CGB1_1F	GTTGGTCTGGAACCCCTCA
	CGB1_2R	ATGGAGCCAATCACAAGAGG

*CGB2*	CGB2_1F	AGGAGAGGCTCACCCCTGAC
	CGB2_2R	CCCGGATAACTTTTCGTATTTTTA

*LHB*	LHB_1F	ATGACATCAAGCGGGTCTAC
	LHB_4R	GGATTAGTGTCCAGGTTACCC

Additional PCR primers tried to amplify *CGB1* and *CGB2* of great apes		
*CGB1*	CGB1_2F	TCTACAGGAAGGATGGCAGAGTG
	CGB1_4R	CTTTGATCTTACGCAGGGTGATG
	CGB1_5R	CATTCTGTTTACCACAGGTGACGA
	CGB1_6R	ATCACAAGAGGCTCATCCCTGAC

*CGB2*	CGB2_3R	CCACCCTCTTTTCTTTTTCTTTCT
	CGB2_5R	ACCGGCTAGGGGAAGAAAAGAAC
	CGB2pr1F	CGAGTAGTGGGACATCCCACTTGCT
	CGB2pr2F	CCTATCAAGGCGTCCTCCCTTTA

Human-specific sequencing primers		
*CGB1*	CGB1_1Fseq	CCTCACCTCAGCTGATCCAC
	CGB1_2Rseq	ACAAGAGGCTCATCCCTGAC

*CGB2*	CGB2_1Fseq	GGAAGGGGAACTGTATCTGAGAG
	CGB2_2Rseq	CCCGGATAACTTTTCGTATTTTT

*LHB*	LHB_1Fseq	ATCAAGCGGGTCTACTCACTCT
	LHB_6Rseq	AGGTTACCCCAGCATCCTATC

All genes^a^	CGBsense_1seqR	TGGTACACCACCCACAAAGA
	CGBsense_2seqF	CTCTTTCTGGAGGAGCGTGA
	CGBsense_2seqR	ACATCGCGGTAGTTGCACA
	CGBsense_3seqF	CCCCTGAGTCTGAGACCTGT
	CGBantisense_1seqR	GTCAACACCACCATCTGTGC
	CGBantisense_2seqF	GTCCAGGAAGCCCTCTGTT
	CGBantisense_2seqR	CACCTTCCACCTCCTTCCAG
	CGBantisense_3seqF	TCCTATTCAGGACCCACCAC

Additional sequencing primers for great apes		
*CGB1*	Chimp-CGB1-3R2-F	GGAAATGTGGATCTACCCTACCT
	Chimp-CGB1_1F1R-F	ATCACAGGTCAAGGGGTGGT
	Chimp-CGB1-2F2-F	AGAGGGAGACCACCCTTCCT

*CGB2*	Gor-CGB2_2F2-F	GAAGGGTCTCTGGGTCTTTGT
	Gor-CGB2-2R2-R	GTCTGGAAGCCGTGTGAGA

*LHB*	Chimp-LHB-1F1R-F	AGCTGGACCCACCCTATGTAT
	Gor-LHB_1F1R-F	CCACCAGAGTTCTGTACTGTGAC
	Orang-LHB_1R2-R	CTCAGCTGTCACTGTGGACTCT
	LHB_3Fseq2-F	ATAGGATGCTGGGGTAACCTG
	LHB_9Rseq-F	GCTTCTGCCCAGTGAGAGAG

F: forward primer; R: reverse primer.

a *CGB2* is transcribed from the sense, and *CGB1*, *LHB* from the antisense strand.

**Table 2:** Nucleotide divergence between species

Gene	Region	Length (bp)^a^	Nucleotide diversity (%)					
			H/C	H/G	H/O	C/G	C/O	G/O
*LHB*	mRNA	1111	1.35	1.44	5.39	1.88	6.29	6.38
	Exons	423	1.42	1.42	4.02	2.84	5.91	5.20
	Introns	588	1.53	1.36	6.12	1.19	6.47	6.81
	5′-UTR	9	0	0	11.11	0	11.11	11.11
	3′-UTR	91	0	2.20	6.45	2.15	6.45	8.60

*CGB1*	mRNA	1366	2.19	2.34	–	2.26	–	–
	Exons	396	0.50	1.51	–	1.49	–	–
	Introns	634	2.68	2.21	–	2.20	–	–
	5′-UTR	174	3.40	4.60	–	3.41	–	–
	3′-UTR	162	3.08	2.47	–	3.08	–	–

*CGB2*	mRNA	1366	1.46	3.00	–	3.80	–	–
	Exons	396	1.26	4.04	–	5.30	–	–
	Introns	634	1.89	3.31	–	3.47	–	–
	5′-UTR	174	1.70	2.30	–	3.97	–	–
	3′-UTR	162	0	1.23	–	1.23	–	–

H: human; C: common chimpanzee; G: gorilla; O: orangutan.

a Length based on human sequence.

**Table 3:** Human *LHB*, *CGB1* and *CGB2* diversity parameters

Gene	Pop	mRNA			Exons			Introns			5′-UTR			3′-UTR		
		*π*^a^ (×10^3^)	*θ*^b^ (×10^3^)	*D*^c^	*π*^a^ (×10^3^)	*θ*^b^ (×10^3^)	*D*^c^	*π*^a^ (×10^3^)	*θ*^b^ (×10^3^)	*D*^c^	*π*^a^ (×10^3^)	*θ*^b^ (×10^3^)	*D*^c^	*π*^a^ (×10^3^)	*θ*^b^ (×10^3^)	*D*^c^
*LHB*	E^d^	3.55	2.82	0.74	3.75	2.31	1.34	4.37	3.65	0.52	0	0	NA	2.3	2.15	0.08
	H^d^	3.09	2.61	0.54	3.69	2.64	0.96	3.87	3.03	0.75	0	0	NA	0.86	2.45	−0.87
	M^d^	3.04	3.28	−0.23	3.58	3.77	−0.13	3.62	3.48	0.11	0	0	NA	1.37	2.50	−0.62
	All	3.92			3.97			4.29			0			1.7		

*CGB1*	E	1.08	1.00	0.18	0.82	0.49	0.78	0.96	1.23	−0.44	2.72	1.12	1.64	0.39	1.21	−0.79
	H	1.31	1.96	−0.98	0.69	0.56	0.31	0.68	1.76	−1.49	5.55	5.13	0.18	0.73	2.76	−1.31
	M	1.73	2.50	−0.95	0.74	0.57	0.40	1.94	2.87	−0.90	0.74	2.62	−1.31	4.42	5.62	−0.49
	All	1.39			0.76			1.15			3.49			1.57		

*CGB2*	E	0.92	0.86	0.16	0.57	0.49	0.18	0.91	1.23	−0.52	0	0	NA	2.8	1.21	1.53
	H	0.90	1.80	−1.45	0.69	2.82	−1.83^*^	0.96	1.76	−1.10	0	0	NA	2.16	1.38	0.76
	M	2.18	3.50	−1.23	2.41	4.02	−1.08	1.65	3.23	−1.39	1.94	2.62	−0.47	3.95	4.21	−0.13
	All	1.26			1.08			1.13			0.53			3.01		

a Estimate of nucleotide diversity per site from average pairwise difference among individuals.

b Estimate of nucleotide diversity per site from number of segregating sites (S).

c Value of Tajima's *D* statistics and significance level: ^*^p < 0.05; NA: not applicable.

d E: Estonians (*n* = 47); H: Chinese Han (*n* = 25); M: Mandenka (*n* = 23).

**Table 4:** Hudson–Kreitman–Aguade (HKA) test results

Population	Gene	*S*^a^	Human vs. chimpanzee			Human vs. gorilla		
			Fixed diff.^b^	HKA^c^*χ*^2^	*p*-Value^d^	Fixed diff.^b^	HKA^c^*χ*^2^	*p*-Value^d^
Estonian	*LHB*	17	15			17		
	*CGB1*	7	35	5.616	0.0178^*^	36	4.492	0.0341^*^
	*CGB2*	6	23	3.469	0.0625	49	8.078	0.0045^**^

Han	*LHB*	14	15			17		
	*CGB1*	12	33	1.701	0.1922	35	0.971	0.3245
	*CGB2*	11	24	0.555	0.4565	48	2.566	0.1092

Mandenka	*LHB*	17	15			16		
	*CGB1*	14	31	1.768	0.1836	32	1.147	0.2841
	*CGB2*	21	21	0.006	0.9367	45	0.883	0.3472

All	*LHB*	24	15			16		
	*CGB1*	22	30	1.740	0.1871	32	1.030	0.3101
	*CGB2*	30	20	0.000	0.9858	41	1.024	0.3116

a Segregating sites = the number of human polymorphisms.

b The number of fixed differences between populations: nucleotide sites at which all of the human sequences are different from the chimpanzee or gorilla sequence.

c *LHB* was used as a reference gene for the HKA test.

d Significance level of HKA *p*-value: ^*^p < 0.05; ^**^p < 0.01.
