# Supplementary material for: The evolution and genomic landscape of CGB1 and CGB2 genes
Source: Mol Cell Endocrinol. 2007 Jan 2;260-262(9):2–11. doi: 10.1016/j.mce.2005.11.049 (PMC2599907; doi:10.1016/j.mce.2005.11.049)
Supplement: Supplementary file 1 [file mmc1.doc]

Supplementary material

**Supplementary Fig.** DNA sequence alignments of (A) *CGB1*; (B) *CGB2*; (C) *LHB* gene in human populations (Estonians, Han and Mandenka) as well as in chimpanzee (Genbank Accession nos. *CGB1* ***DQ238547***, *CGB2* ***DQ238549***, *LHB* ***DQ238551***), gorilla (Genbank Accession nos. *CGB1* ***DQ238548***, *CGB2* ***DQ238550***, *LHB* ***DQ238552***) and orangutan (Genbank Accession no. *LHB* ***DQ238553***). Polymorphic positions in humans are marked with N.

*one orangutan *LHB* sequence originates from Maston and Ruvolo (2002), Genbank Accession No. ***AF397592.***

**Supplementary Table.** Single nucleotide polymorphisms identified in human (H) *CGB1* (***ss48399944 - ss48399963***)*,* *CGB2* (***ss48399964 - ss48399997***) and *LHB* (***ss48399882 - ss48399908***) genes located according to predicted transcription start site; and ancestral alleles detected at these positions in chimpanzee (C), gorilla (G) and orangutan (O).

CGB1_est 1 CCCCAGGGCCAGTGAGGGCCCTGCGTTCCGTGGCGCCCCCTGGAGGGAGGAAGGGGAACT

CGB1_han 1 ............................................................

### Supplementary Figure (A)

CGB1_man 1 ............................................................

CGB1_chimp 1 .......................T....................................

CGB1_gorilla 1 .......................T.....C..............................

CGB1_est 61 GCATCTGAGAGAGA--GCAGCCAATTGGGTCCGCTGACTCNGGCCAGGTTCCCGTGCCGC

CGB1_han 61 ..............--.................N........N.................

CGB1_man 61 ..............--...............N............................

CGB1_chimp 61 .T............GA........................C....G..............

CGB1_gorilla 61 .T............--......C.................C....G........C.....

CGB1_est 119 GTCCAACACCCCTCACTCCCTGTCTCACTCCCCCACGGAGACTCAATTTACTTTCCATGT

CGB1_han 119 ......................................................N.....

CGB1_man 119 ............................................................

CGB1_chimp 121 ............................................................

CGB1_gorilla 119 ..........................C.................................

CGB1_est 179 CCACATTCCCAGTGCTTGCGGAAGATATCCCGCTAAGAGAGAGACATGTCAAAGGTAGGG

CGB1_han 179 ............................................................

CGB1_man 179 ............................................................

CGB1_chimp 181 ............................................................

CGB1_gorilla 179 ............................................................

CGB1_est 239 TAGATCCACATTTCCGGGCACCAAAGATGGAGATGTTCCAGGAAAGACTGCAGGGCCCCT

CGB1_han 239 .................................N..........................

CGB1_man 239 .................................N..........................

CGB1_chimp 241 ...............A............................................

CGB1_gorilla 239 ..C............A............................................

CGB1_est 299 GGGCACCTTCCACCTCCTTCCAGGCCATCACTGGCATGAGAAGGGGCAGACCCGTGTGAG

CGB1_han 299 ............................................................

CGB1_man 299 ............................................................

CGB1_chimp 301 ....................................................A.C.....

CGB1_gorilla 299 ....................................................A.......

CGB1_est 359 CTGTGGAAGGAGGCCTCTTTCTGGAGGAGCGTGACCCCCAGTAAGCTTCAGGTGGGTCAG

CGB1_han 359 ..................................N.........................

CGB1_man 359 ..................................N.....................N...

CGB1_chimp 361 .G...............................T......................G...

CGB1_gorilla 359 ........................................................G...

CGB1_est 419 TTCCTGAGGGTGCGGATCTGAAATGTTGGGGTATCTCAGGTCCTCTGGGCTGTGGGGTGG

CGB1_han 419 ............................................................

CGB1_man 419 ............................................................

CGB1_chimp 421 ............G...............................................

CGB1_gorilla 419 ............G...............................................

CGB1_est 479 GCTCTGAAAGGCAGGTGTCCGGGTGGTGGGTCCTGAATAGAAGATGCCGGGAAGGGTCTC

CGB1_han 479 ............................................................

CGB1_man 479 ............................................................

CGB1_chimp 481 ...................G....................G...................

CGB1_gorilla 479 ...................G....................G...................

CGB1_est 539 TGGGTCTTTGTGGGTGGTGTACCATGCGGGATGGGAAGGCCAGGACTGGGGGCTGCAGTC

CGB1_han 539 ............................................................

CGB1_man 539 ............................................................

CGB1_chimp 541 .............T..........C......................C........G...

CGB1_gorilla 539 ........................C......................C........G...

CGB1_est 599 TCAGACCNGGGTGAAGCAGTGTCCTTGNCCCAGNGGCTGCTGCTGTTGCTGCTGCTGAGC

CGB1_han 599 .......C....................................................

CGB1_man 599 .......C....................................................

CGB1_chimp 601 .......C...................C.....G..........................

CGB1_gorilla 599 .......C...................C.....G..........................

CGB1_est 659 ATGGGCGGGACATGGGCATCCAAGGAGCCGCTTCGGCCACGGTGCCGCCCCATCAAT-GC

CGB1_han 659 .........................................................-..

CGB1_man 659 .........................................................-..

CGB1_chimp 661 .........................................................-..

CGB1_gorilla 659 .........................................................T..

### Supplementary Figure (A) cont.

CGB1_est 718 CACCCTGGCTGTGGAGAAGGAGGGCTGCCCCGTGTGCATCACCGTCAACACCACCATCTG

CGB1_han 718 ............................................................

CGB1_man 718 ............................................................

CGB1_chimp 720 ............C....................................G..........

CGB1_gorilla 719 ............C...............................................

CGB1_est 778 TGCCGGCTACTGCCCCACCATGGTGAGCTGCCCGGGGCCNGGGCAGGTGCTGCCACCTCA

CGB1_han 778 .......................................A....................

CGB1_man 778 ....................................N.......................

CGB1_chimp 780 .......................................G....................

CGB1_gorilla 779 ...................................A...G....................

CGB1_est 838 GGGCCAGACCCACAGAGGCAGCGGGGGAGGAAGGGTGGTCTGCCTCTCTGGTCAGGGGCT

CGB1_han 838 ............................................................

CGB1_man 838 ............................................................

CGB1_chimp 840 ............G..........T.................C..................

CGB1_gorilla 839 ......................C.....................................

CGB1_est 898 GCGGAATGGGGTGTGGGAGGGCAGGAACAGAGGGCTTCCTGGACCCCTGAGTCTGAGACC

CGB1_han 898 .......................................N....................

CGB1_man 898 ...........................................................N

CGB1_chimp 900 ................................A......G....T...............

CGB1_gorilla 899 ............................................T...............

CGB1_est 958 TGTGGGGGCAGCTGGGGAGCTCAGCTGAGGCGCTGGCCCCAGGCACATGCTCATTCNCCC

CGB1_han 958 ..........N.............................................C...

CGB1_man 958 ........................................................C...

CGB1_chimp 960 ........................................................C...

CGB1_gorilla 959 .................................C.............A........C...

CGB1_est 1018 ACTCACACGGCTTCCAGACCCGCGTGCTGCAGGGGGTCCTGCCGGCCCTGCCTCAGGTGG

CGB1_han 1018 ............................................................

CGB1_man 1018 ............................................................

CGB1_chimp 1020 ............................................................

CGB1_gorilla 1019 ............................................C..G............

CGB1_est 1078 TGTGCAACTACCGCGATGTGCGCTTCGAGTCCATCCGGCTCCCTGGCTGCCCGCGCGGCG

CGB1_han 1078 ............................................................

CGB1_man 1078 ...................................N........................

CGB1_chimp 1080 ............................................................

CGB1_gorilla 1079 ............................................................

CGB1_est 1138 TGAACCCCGTGGTCTCCTACGCCGTGGCTCTCAGCTGTCAATGTGCACTCTGCCGCCGCA

CGB1_han 1138 ............................................................

CGB1_man 1138 ............................................................

CGB1_chimp 1140 ............................................................

CGB1_gorilla 1139 ................................................G..........G

CGB1_est 1198 GCACCACTGACTGCGGGGGTCCCAAGGACCACCCCTTGACCTGTGATGACCCCCGCTTCC

CGB1_han 1198 ............................................................

CGB1_man 1198 ..................N.........................................

CGB1_chimp 1200 ............................................................

CGB1_gorilla 1199 ...........................................C................

CGB1_est 1258 AGGACTCCTCTTCCTCAAAGGCCCCTCCCCCCAGCCTTCCAAGTCCATCCCGTCTCNCGG

CGB1_han 1258 ....................................................N...C...

CGB1_man 1258 ...N................................................N...C...

CGB1_chimp 1260 ...C.......................................C........A...CT..

CGB1_gorilla 1259 ...C.......................................C........A...C...

CGB1_est 1318 GGCCCTAGGACACCCCGATCCTCCCACAATAAAGGCTTCTCAATCCGCA

CGB1_han 1318 ......N..........................................

CGB1_man 1318 ......N..........................................

CGB1_chimp 1320 A.....CA....T....................................

CGB1_gorilla 1319 ......CA.....................C...................

CGB2_est 1 CCCCAGGGCCAGTGAGGGCCCTGCGTTCCGTGGCGCCCCCTGGAGGGAGGAAGGGGAACT

### Supplementary Figure (B)

CGB2_han 1 ............................................................

CGB2_man 1 ............................................................

CGB2_chimp 1 .......................T....................................

CGB2_gorilla_1 1 .............................A..............................

CGB2_gorilla_2 1 .............................A..............................

CGB2_est 61 GTATCTGAGAGAGAG--CAGCCAATTGGGTCCGCTGACTCCGGCCGGGTTCCCGTGCCGC

CGB2_han 61 ...............--...........................................

CGB2_man 61 ...............--...........................................

CGB2_chimp 61 ...............AG...........................................

CGB2_gorilla_1 61 ...............--............G..........G...................

CGB2_gorilla_2 61 ...............--............G..........G...................

CGB2_est 119 GTCCAACACCCCTCACTCCCTGTCTCACTCCCCCACGGAGACTCAATTTACTTTCCATGT

CGB2_han 119 ............................................................

CGB2_man 119 .......................................N.N..................

CGB2_chimp 121 ............................................................

CGB2_gorilla_1 119 ..............G.............................................

CGB2_gorilla_2 119 ..............G.............................................

CGB2_est 179 CCACATCCCCAGTGCTTGCGGAAGATATCCCGCTAAGAGAGAGACATGTCAAAGGTAGGG

CGB2_han 179 ............................................................

CGB2_man 179 ............................................................

CGB2_chimp 181 ......T...........T.........................................

CGB2_gorilla_1 179 ............................................................

CGB2_gorilla_2 179 ............................................................

CGB2_est 239 TAGATCGACATTTCCAGGCACCAAAGATGGAGATGTTCCAGGAAAGACTGCAGGGCCCCT

CGB2_han 239 ............................................................

CGB2_man 239 ......N.....................................................

CGB2_chimp 241 ......C.....................................................

CGB2_gorilla_1 239 ......C.................G.................T.................

CGB2_gorilla_2 239 ......C.................G.................T.................

CGB2_est 299 GGGCACCTTCCACCTNCTTCCAGGCCATCACTGGCATGAGAAGGGGCAGACCAGTGTGAG

CGB2_han 299 ...............G....................................N.......

CGB2_man 299 ...............G............................................

CGB2_chimp 301 ...............C............................................

CGB2_gorilla_1 299 .............G.C....................................C.......

CGB2_gorilla_2 299 .............G.C....................................C.......

CGB2_est 359 CTGNGGAAGGAGGCCTCTTTCTGGAGGAGCGTGACCCCCAGTAAGCTTCAGGTGGGGCAG

CGB2_han 359 .........N..................................................

CGB2_man 359 .........N.....N...................N........................

CGB2_chimp 361 ...T................................T.......................

CGB2_gorilla_1 359 ...T...................................G....................

CGB2_gorilla_2 359 ...T...................................G....................

CGB2_est 419 TTCCTGAGGGTGGGGATCTAAAATGTTGGGGTATCTGAGATCCTCTGGGCTGTGGGGTGG

CGB2_han 419 ............................................................

CGB2_man 419 ............................................................

CGB2_chimp 421 ...................G................C..G....................

CGB2_gorilla_1 419 ...................G................C..G....................

CGB2_gorilla_2 419 ...................G................C..G....................

CGB2_est 479 GCTCTGAAAGGCAGGTGTCCGGGTGGTGGGTCCTGAATAGGAGATGCCACGAAGGGTCTC

CGB2_han 479 ............................................................

CGB2_man 479 ................................................NN..........

CGB2_chimp 481 ...................G............................GG..........

CGB2_gorilla_1 479 ...................G............................GG..........

CGB2_gorilla_2 479 ...................G............................GG..........

CGB2_est 539 TGGGTCTTTGTGGGTGGTGTACCACGNGGGATGGGAAGGCCAGGACTCGGGGCTGCNGTC

CGB2_han 539 ........................N.C.............................G...

CGB2_man 539 ..........................C.............................G...

CGB2_chimp 541 ..........................C............................TG...

CGB2_gorilla_1 539 ..........................C.................G...A......TGC..

CGB2_gorilla_2 539 ..........................C.................G...A......TGC..

CGB2_est 599 TCAGACCCGGGTGAAGCAGTGTCCTTGTCCCAGGGGCTGCTGCTGTTGCTGCTGCTGAGC

CGB2_han 599 ............................................................

### Supplementary Figure (B) cont.

CGB2_man 599 ............................................................

CGB2_chimp 601 .......................................A....................

CGB2_gorilla_1 599 A........................C.C...........------------.........

CGB2_gorilla_2 599 A........................C.C...........------------.........

CGB2_est 659 ATGGGCGGGACATGGGCATCCAAGGAGCCGCTTCGGCCACGGTGCCNCCCCATCAATGCC

CGB2_han 659 ...........................NN.................G.............

CGB2_man 659 ......................N....NN..........N....................

CGB2_chimp 661 .....T........................................G.............

CGB2_gorilla_1 647 ......................G....A..................G.............

CGB2_gorilla_2 647 ......................G....A..................G.............

CGB2_est 719 ACCCTGGCTGTGGAGAAGGAGGGCTGCCCCGTGTGCATCACCGTCAACACCACCATCTGT

CGB2_han 719 ............................................................

CGB2_man 719 ............................................................

CGB2_chimp 721 ...........C................................................

CGB2_gorilla_1 707 ...T.......C................................................

CGB2_gorilla_2 707 ...T.......C................................................

CGB2_est 779 GCCGGCTACTGCCCCACCATGGTGAGCTGCCCGGGGCCGGGGCAGGTGCTGCCACCTCAG

CGB2_han 779 ......................................N.....................

CGB2_man 779 ............................................................

CGB2_chimp 781 ............................................................

CGB2_gorilla_1 767 ............................................................

CGB2_gorilla_2 767 ............................................................

CGB2_est 839 GGCCAGACCCACAGAGGCAGCGGGGGAGGAAGGGTGGTCTGCCTCTCTGGTCAGGGGCTG

CGB2_han 839 ............................................................

CGB2_man 839 .................................N..........................

CGB2_chimp 841 ....................T...................C...................

CGB2_gorilla_1 827 .....................C......................................

CGB2_gorilla_2 827 .....................C......................................

CGB2_est 899 CGGAATGGGGTGTGGGAGGGCAGGAACAGAGGGCTTCCTGGACCCCTGAGTCTGAGACCT

CGB2_han 899 ............................................................

CGB2_man 899 .....................N................N.....................

CGB2_chimp 901 ...............................A......G....T................

CGB2_gorilla_1 887 .....C..............................................--......

CGB2_gorilla_2 887 ....................................................--......

CGB2_est 959 GTGGGGGCAGCTGGGGAGCTCAGCTGAGGCGCTGGCCCCAGGCACATGCTCATTCTCCCA

CGB2_han 959 ............................................................

CGB2_man 959 ............................................................

CGB2_chimp 961 .......................................................C....

CGB2_gorilla_1 945 ........................C..............................C...T

CGB2_gorilla_2 945 ........................C..............................C...T

CGB2_est 1019 CTCACACGGCTTCCAGACCCGCGTGCTGCAGGGGGTCCTGCCGGCCCTGCCTCAGGTGGT

CGB2_han 1019 ............................................................

CGB2_man 1019 ............................................................

CGB2_chimp 1021 ............................................................

CGB2_gorilla_1 1005 ...........................................C.......C........

CGB2_gorilla_2 1005 ...........................................C.......C........

CGB2_est 1079 GTGCAACTACCGCGATGTGCGCTTCGAGTCCATCCGGCTCCCTGGCTGCCCGCGCGGCGT

CGB2_han 1079 ............................................................

CGB2_man 1079 ......................N.....................................

CGB2_chimp 1081 ............................................................

CGB2_gorilla_1 1065 ............................................................

CGB2_gorilla_2 1065 ............................................................

CGB2_est 1139 GAACCCCGTGGTCTCCTACGCCGTGGCTCTCAGCTGTCAATGTGCACTCTGCCGCCGCAG

CGB2_han 1139 .....N................................NN....................

CGB2_man 1139 .......N....................................................

CGB2_chimp 1141 ............................................................

CGB2_gorilla_1 1125 ............................................................

CGB2_gorilla_2 1125 ............................................................

CGB2_est 1199 CACCACTGACTGCGGGGGTCCCAAGGACCACCCCTTGACCTGTGATGACCCCCGCTTCCA

CGB2_han 1199 ............................................................

### Supplementary Figure (B) cont.

CGB2_man 1199 ............N...............................................

CGB2_chimp 1201 ............................................................

CGB2_gorilla_1 1185 ..............................................C.............

CGB2_gorilla_2 1185 ..............................................C.............

CGB2_est 1259 GGNCTCCTCTTCCTCAAAGGCCCCTCCCCCCAGCCTTCCAAGCCCATCCCGACTCCCGGG

CGB2_han 1259 ............................................................

CGB2_man 1259 .....................N......................................

CGB2_chimp 1261 ..C.........................................................

CGB2_gorilla_1 1245 ..C.......................................T.................

CGB2_gorilla_2 1245 ..C.......................................T.................

CGB2_est 1319 GCCCTCAGACACCCCGATCCTCCCACAATAAAGGCTTCTCAATCCGCA

CGB2_han 1319 ................................................

CGB2_man 1319 ................................................

CGB2_chimp 1321 ................................................

CGB2_gorilla_1 1305 ................................................

CGB2_gorilla_2 1305 ................................................

LHB_est 1 GCACCAAGGATGGAGATGCTCCAGGTAAGACTNCAGGGCCCCTGGGCACCTTCCACCTCC

### Supplementary Figure (C)

LHB_han 1 ............................................................

LHB_man 1 ............................................................

LHB_chimp_1 1 ................................G...........................

LHB_chimp_2 1 ................................G...........................

LHB_gorilla_1 1 ................................G...........................

LHB_gorilla_2 1 ................................G...........................

LHB_orang_1 1 A...............................G...........................

LHB_orang_2 1 A...............................G...........................

LHB_orangutan* 1 ------------------------------------........................

LHB_est 61 TTCCAGGCCATCACTGGCATGAGAAGGGGCAGACCCGTGTGAGCTGTGGAAGGAGGCCTC

LHB_han 61 ............................................................

LHB_man 61 ...................................N........................

LHB_chimp_1 61 .................T..........................................

LHB_chimp_2 61 ............................................................

LHB_gorilla_1 61 ............................................................

LHB_gorilla_2 61 ...................................A........................

LHB_orang_1 61 C....................-------................G...............

LHB_orang_2 61 C....................-------................G...............

LHB_orangutan* 25 C....................-------................G...............

LHB_est 121 TTTCTGGAGGNGCNTGACCCCCAGTAAGCTTCAGGTGGGGCANTTCCTGAGGGTGGGGAT

LHB_han 121 ..........................................G.................

LHB_man 121 ..........................................G.................

LHB_chimp_1 121 ..........G..A............................G.................

LHB_chimp_2 121 ..........G..A............................G.................

LHB_gorilla_1 121 ..........G..A............................G.................

LHB_gorilla_2 121 ..........G..A............................G.................

LHB_orang_1 114 ......A...A..G............................G.................

LHB_orang_2 114 ......A...A..G............................G.................

LHB_orangutan* 78 ......A...A..G............................G.................

LHB_est 181 CTGAAATGTTGGGGCATCTCAGGTCCTCTGGGCTGTGGGGTGGGCTCNGAAAGGCAGGTG

LHB_han 181 ...............................................T............

LHB_man 181 ...............................................T............

LHB_chimp_1 181 ..............T................................T............

LHB_chimp_2 181 ..............T................................T............

LHB_gorilla_1 181 ...............................................T............

LHB_gorilla_2 181 ...............................................T............

LHB_orang_1 174 ..............T.....G...................G......T............

LHB_orang_2 174 ..............T.....G..........................T............

LHB_orangutan* 138 ..............T.....G...................G......T............

LHB_est 241 TCCGGGTGGTGGGTNCTGAATAGGAGATGCCNNGAAGGGTCTCTGGGTCTTTGTGGGTGG

LHB_han 241 ..............C.............................................

LHB_man 241 ..............C................AG...........................

LHB_chimp_1 241 ..A...........C................AG...........................

LHB_chimp_2 241 ..A...........C................AG...........................

LHB_gorilla_1 241 ..............C................AG...........................

LHB_gorilla_2 241 ..............C................AG...........................

LHB_orang_1 234 ...A..........C................GG...............G.C.........

LHB_orang_2 234 ...A..........C................GG...............G.C.........

LHB_orangutan* 198 ...A..........C................GG...............G.C.........

LHB_est 301 TGTACCACGCGGGATGGGAAGGCCAGGACTCGGGGCTGCGGTCTCAGACCNGGGTGAAGC

LHB_han 301 ............................................................

LHB_man 301 .......N...............N....................................

LHB_chimp_1 301 ...........................G...A..................T.........

LHB_chimp_2 301 ...........................G...A..................T.........

LHB_gorilla_1 301 ...........................G...A......T...........C.........

LHB_gorilla_2 301 ...........................G..........T...........C.........

LHB_orang_1 294 ......C..G.................G...A.......A..........C.........

LHB_orang_2 294 ......C..G.................G...A.......A..........C.........

LHB_orangutan* 258 ......C..G.................G...A.......A..........C.........

LHB_est 361 AGTGTCCTTGTCCCAGGGGCTGCTGCTGTTGCTGCTGCTGAGCATNGGCGGGGCATGGGC

LHB_han 361 .............................................G..............

### Supplementary Figure (C) cont.

LHB_man 361 .............................................G......N.......

LHB_chimp_1 361 ..........C..................................G..T...........

LHB_chimp_2 361 ..........C..................................G..T...........

LHB_gorilla_1 361 ..........C...........T......................G..............

LHB_gorilla_2 361 ..........C..................................G..............

LHB_orang_1 354 ...............................A.............G......A.......

LHB_orang_2 354 ...............................A.............G......A.......

LHB_orangutan* 318 ...............................A.............G......A.......

LHB_est 421 ATCCAGGGAGCCGCTTCGGCCANGGTGCCACCCCATCAATGCCATCCTGGCTGTNGAGAA

LHB_han 421 ............................................................

LHB_man 421 .....................N.......N..............................

LHB_chimp_1 421 ............A.........T.....................C.........C.....

LHB_chimp_2 421 ............A.........T.....................C.........C.....

LHB_gorilla_1 421 ......................C......G..............C.........C.....

LHB_gorilla_2 421 ......................C......G..............C.........C.....

LHB_orang_1 414 .....A................C......G..............C.........C.....

LHB_orang_2 414 .....A................C......G..............C.........C.....

LHB_orangutan* 378 .....A................C......G..............C.........C.....

LHB_est 481 GGAGGGCTGCCCNGTGTGCATCACCGTCAACACCACCATCTGTGCCGGCTACTGCCCCAC

LHB_han 481 .............................................N..............

LHB_man 481 ............................................................

LHB_chimp_1 481 ............C...............................................

LHB_chimp_2 481 ............C...............................................

LHB_gorilla_1 481 ............C...............................................

LHB_gorilla_2 481 ............C...............................................

LHB_orang_1 474 ............C...............................................

LHB_orang_2 474 ............C...............................................

LHB_orangutan* 438 ............C......G........................................

LHB_est 541 CATGGTGAGCTGCCNGGGGCCNGGGGCAGATGCTGCCACCTCAGGGCCAGACCCACAGAG

LHB_han 541 ............................................................

LHB_man 541 ............................................................

LHB_chimp_1 541 ..............T......A.....G................................

LHB_chimp_2 541 ..............T......A.....G................................

LHB_gorilla_1 541 ..............T......A.....G................................

LHB_gorilla_2 541 ..............T......A.....G................................

LHB_orang_1 534 ..............T......A...-...G..............................

LHB_orang_2 534 ..............T......A...-...G..............................

LHB_orangutan* 498 ..............T......A...-...G..............................

LHB_est 601 GCAGCGGGGGAGGAAGGGTGGTCTGCCTCTCTGGCCTGCGGTTGGGGAATGGGGTGTGGG

LHB_han 601 ............................................................

LHB_man 601 ............................................................

LHB_chimp_1 601 ............................................................

LHB_chimp_2 601 ............................................................

LHB_gorilla_1 601 ......................................T.....................

LHB_gorilla_2 601 ......................................T.....................

LHB_orang_1 593 ..........C....A..............G.............................

LHB_orang_2 593 ..........C....A..............G.............................

LHB_orangutan* 557 ...............A..............G.............................

LHB_est 661 AAGGCAGGAACAGAGGGCTTCCTGGGCTCCTGAGTCCAGGACCTGTGGGGTCAGCTTGGG

LHB_han 661 ............................................................

LHB_man 661 ............................................................

LHB_chimp_1 661 .G.........................A................................

LHB_chimp_2 661 .G.........................A................................

LHB_gorilla_1 661 .G.........................A................................

LHB_gorilla_2 661 .G.........................A................................

LHB_orang_1 653 .G.......................A.A........T..A................G...

LHB_orang_2 653 .G.......................A.A........T..A................G...

LHB_orangutan* 617 .G.......................A.A........T..A................G...

LHB_est 721 AGCTCAGCTGAGGCGCTGGCCTCAGGCACATGCTCATTCCCCCACTCACACGGCCTCCAG

LHB_han 721 ............................................................

LHB_man 721 ............................................................

LHB_chimp_1 721 ......................................................A.....

LHB_chimp_2 721 ......................................................A.....

LHB_gorilla_1 721 .......................C....................................

### Supplementary Figure (C) cont.

LHB_gorilla_2 721 .......................C....................................

LHB_orang_1 713 .....................C....................-..C..A.....T.....

LHB_orang_2 713 .....................C....................-..C..A.....T.....

LHB_orangutan* 677 .....................C....................-..C..A.....T.....

LHB_est 781 ATGCGCGTGCTGCAGGCGGTCCTGCCGCCCCTGCCTCAGGTGGTGTGCACCTACCGTGAT

LHB_han 781 ............................................................

LHB_man 781 ............................................................

LHB_chimp_1 781 ............................................................

LHB_chimp_2 781 ............................................................

LHB_gorilla_1 781 ................GT..........................................

LHB_gorilla_2 781 ................GT..........................................

LHB_orang_1 772 .C.............T............................A...........C..C

LHB_orang_2 772 .C.............T............................A...........C..C

LHB_orangutan* 736 .C.............T............................A..T........C..C

LHB_est 841 GTGCGCTTCGAGTCCATCCGGCTCCCTGGCTGCCCGCGTGGNGTGGACCCCGTGGTCTCC

LHB_han 841 ............................................................

LHB_man 841 ............................................................

LHB_chimp_1 841 ...................................A.....C..................

LHB_chimp_2 841 ...................................A.....C..................

LHB_gorilla_1 841 ...................A.....................C.........A........

LHB_gorilla_2 841 .........................................C.........A........

LHB_orang_1 832 ..............T...T......................T..................

LHB_orang_2 832 ..............T...T......................T..................

LHB_orangutan* 796 ..............T...T......................T..................

LHB_est 901 TTCCCTGTGGCTCTCAGCTGTCGCTGTGGACCCTGCCGCCGCAGCACCTCTGACTGTGGG

LHB_han 901 ............................................................

LHB_man 901 ............................................................

LHB_chimp_1 901 ...........C................................................

LHB_chimp_2 901 ...........C................................................

LHB_gorilla_1 901 .....................................A......................

LHB_gorilla_2 901 .....................................A......................

LHB_orang_1 892 .A.G.C................A........T............................

LHB_orang_2 892 .A.G.C................A........T............................

LHB_orangutan* 856 .A.G.C................A........T............................

LHB_est 961 GGTCCCAAAGACCACCCCTTGACCTGTGACCACCCCCAACTCTCAGGCCTCCTCTTCCTC

LHB_han 961 ............................................................

LHB_man 961 ............................................................

LHB_chimp_1 961 ........................................................T...

LHB_chimp_2 961 ........................................................T...

LHB_gorilla_1 961 ........C...................................................

LHB_gorilla_2 961 ........C...................................................

LHB_orang_1 952 ........G.................................C.................

LHB_orang_2 952 ........G.................................C.................

LHB_orangutan* 916 ........G.................................C.................

LHB_est 1021 TAAAGACCCTCCCCGCAGCCTTCCAAGTCCATCCCGACTCCTGGAGCCCT--GACACCCC

LHB_han 1021 ..................................................--........

LHB_man 1021 ..................................................--........

LHB_chimp_1 1021 ..................................................--........

LHB_chimp_2 1021 ..................................................--........

LHB_gorilla_1 1021 ..............C............C......................--........

LHB_gorilla_2 1021 ..............C............C......................--........

LHB_orang_1 1012 .............T....................................CA..T.....

LHB_orang_2 1012 .............T....................................CA..T.....

LHB_orangutan* 976 .............T....................................CA..T.....

LHB_est 1079 GATCCTCCCACAATAAAGGCTTCTCAATCNGCA

LHB_han 1079 .................................

LHB_man 1079 .................................

LHB_chimp_1 1079 .............................C...

LHB_chimp_2 1079 .............................C...

LHB_gorilla_1 1079 .............................C...

LHB_gorilla_2 1079 ...T.........................C...

LHB_orang_1 1072 .T...............A...........C...

LHB_orang_2 1072 .T...............A...........C...

LHB_orangutan* 1036 .T...----------------------------

**Supplementary Table.** Single nucleotide polymorphisms identified in human (H) *CGB1,* *CGB2* and *LHB* genes located according to predicted transcription start site; and ancestral alleles detected at these positions in chimpanzee (C), gorilla (G) and orangutan (O).

*causes a non-synonymous change

|  | CGB1 | | | |  | CGB2 | | | |  | LHB | | | | |
| --- | --- | --- | --- | --- | --- | --- | --- | --- | --- | --- | --- | --- | --- | --- | --- |
| position | | H | C | G | position | | H | C | G | position | | H | C | G | O |
| 5'UTR | 89 | C/T | C | C |  | 158 | G/C | G | G |  |  |  |  |  |  |
|  | 91 | C/A | C | C |  | 160 | C/A | C | C |  |  |  |  |  |  |
|  | 98 | G/T | C | C |  |  |  |  |  |  |  |  |  |  |  |
|  | 100 | G/T | G | G |  |  |  |  |  |  |  |  |  |  |  |
|  | 172 | C/A | C | C |  |  |  |  |  |  |  |  |  |  |  |
| Intron 1 | 272 | T/A | T | T |  | 246 | G/C | C | C |  | 33 | A/G | G | G | G |
|  | 393 | C/G | C | C |  | 314 | G/C | C | C |  | 96 | C/T | C | A/C | C |
|  | 415 | T/G | G | G |  | 351 | A/C | C | C |  | 131 | G/A | G | G | A |
|  | 606 | C/T | C | C |  | 362 | T/C | T | T |  | 134 | A/G | A | A | G |
|  | 626 | C/T | C | C |  | 368 | G/A | G | G |  | 163 | G/T | G | G | G |
|  |  |  |  |  |  | 374 | T/G | T | T |  | 228 | T/C | T | T | T |
|  |  |  |  |  |  | 394 | C/T | C | C |  | 255 | C/A | C | C | C |
|  |  |  |  |  |  | 527 | A/G | G | G |  | 272 | A/G | A | A | G |
|  |  |  |  |  |  | 528 | C/G | G | G |  | 273 | G/C | G | G | G |
|  |  |  |  |  |  | 563 | C/T | C | C |  | 308 | C/T | C | C | C |
|  |  |  |  |  |  | 565 | C/T | C | C |  | 324 | C/T | C | C | C |
|  |  |  |  |  |  | 595 | G/A | G | G |  | 351 | C/T | T | C | C |
| Exon 2 | 632* | A/G | G | G |  | 681 | A/G | G | G |  | 406 | G/A | G | G | G |
|  |  |  |  |  |  | 686* | C/A | A | A |  | 413 | G/A | G | G | A |
|  |  |  |  |  |  | 687* | C/T | C | C |  | 442 | A/T | A | A | A |
|  |  |  |  |  |  | 698* | C/T | C | C |  | 443* | T/C | T | C | C |
|  |  |  |  |  |  | 705 | G/A | G | G |  | 450* | A/G | A | G | G |
|  |  |  |  |  |  |  |  |  |  |  | 475 | C/G | C | C | C |
|  |  |  |  |  |  |  |  |  |  |  | 493* | C/A | C | C | C |
|  |  |  |  |  |  |  |  |  |  |  | 526* | C/T | C | C | C |
| Intron 2 | 814 | G/A | G | G |  | 816 | G/C | G | G |  | 555 | T/C | T | T | T |
|  | 817 | A/G/C | G | G |  | 872 | G/A | G | G |  | 562 | -/A | A | A | A |
|  | 937 | T/G | G | T |  | 920 | A/T | A | A |  |  |  |  |  |  |
|  | 957 | C/G | C | C |  |  |  |  |  |  |  |  |  |  |  |
|  | 968 | G/A | G | G |  |  |  |  |  |  |  |  |  |  |  |
|  | 1014 | C/T | C | C |  |  |  |  |  |  |  |  |  |  |  |
| Exon 3 |  |  |  |  |  | 1101* | T/A | T | T |  | 882 | C/T | C | C | T |
|  |  |  |  |  |  | 1144 | C/G | C | C |  |  |  |  |  |  |
|  |  |  |  |  |  | 1146* | G/A | G | G |  |  |  |  |  |  |
|  |  |  |  |  |  | 1177 | A/G | A | A |  |  |  |  |  |  |
|  |  |  |  |  |  | 1178* | A/C | A | A |  |  |  |  |  |  |
| 3'UTR | 1216 | G/T | G | G |  | 1211 | C/T | C | C |  | 1108 | C/T | C | C | C |
|  | 1261 | A/C | C | C |  | 1261 | A/C | C | C |  |  |  |  |  |  |
|  | 1310 | T/A | A | A |  | 1280 | C/A | C | C |  |  |  |  |  |  |
|  | 1314 | C/T | C | C |  |  |  |  |  |  |  |  |  |  |  |
|  | 1324 | A/C | C | C |  |  |  |  |  |  |  |  |  |  |  |
